# Solvent-Guided Fractionation of Green *Coffea arabica* Seeds Uncovers Divergent Antioxidant and Mitochondria-Targeted Activities

**DOI:** 10.3390/plants15101494

**Published:** 2026-05-13

**Authors:** Grațiana Ruse, Ștefana Avram, Andreea-Maria Munteanu, Oana-Andrada Iftode, Laurian Vlase, Ana-Maria Vlase, Delia Muntean, Alexandra Mioc, Raluca Pop, Alina-Arabela Jojic, Codruța-Marinela Șoica, Diana-Simona Tchiakpe-Antal

**Affiliations:** 1 Discipline of Pharmaceutical Botany, Faculty of Pharmacy, “Victor Babeș” University of Medicine and Pharmacy, 2 Eftimie Murgu Square, 300041 Timișoara, Romania; gratiana.ruse@umft.ro (G.R.); alina.jojic@umft.ro (A.-A.J.); diana.antal@umft.ro (D.-S.T.-A.); 2 Discipline of Pharmacognosy-Phytotherapy, Faculty of Pharmacy, “Victor Babeş” University of Medicine and Pharmacy, 300041 Timişoara, Romania; stefana.avram@umft.ro; 3 Research and Processing Center for Medicinal and Aromatic Plants, Faculty of Pharmacy, “Victor Babeș” University of Medicine and Pharmacy, 2 Eftimie Murgu Square, 300041 Timișoara, Romania; 4 Department of Pharmaceutical Chemistry and Biochemistry, Faculty of Pharmacy, “Victor Babeș” University of Medicine and Pharmacy, 2 Eftimie Murgu Square, 300041 Timișoara, Romania; 5 Research Center for Experimental Pharmacology and Drug Design, Faculty of Pharmacy, “Victor Babeș” University of Medicine and Pharmacy, 2 Eftimie Murgu Square, 300041 Timișoara, Romania; alexandra.mioc@umft.ro (A.M.); codrutasoica@umft.ro (C.-M.Ș.); 6 Research Center for Pharmaco-Toxicological Evaluations, Faculty of Pharmacy, “Victor Babeș” University of Medicine and Pharmacy, 2 Eftimie Murgu Square, 300041 Timișoara, Romania; 7 University Clinic of Toxicology, Drug Industry, Management, Marketing and Dermatopharmacy, Faculty of Pharmacy, “Victor Babeș” University of Medicine and Pharmacy, 2 Eftimie Murgu Square, 300041 Timișoara, Romania; 8 Department of Pharmaceutical Technology and Biopharmaceutics, Faculty of Pharmacy, “Iuliu Hațieganu” University of Medicine and Pharmacy, 8 Victor Babeș Street, 400012 Cluj-Napoca, Romania; laurian.vlase@umfcluj.ro; 9 Discipline of Pharmaceutical Botany, Faculty of Pharmacy, “Iuliu Hațieganu” University of Medicine and Pharmacy, 400012 Cluj-Napoca, Romania; gheldiu.ana@umfcluj.ro; 10 Discipline of Microbiology-Virusology, Faculty of Medicine, “Victor Babeș” University of Medicine and Pharmacy, 2 Eftimie Murgu Square, 300041 Timișoara, Romania; muntean.delia@umft.ro; 11 Discipline of Pharmacology, Physiology and Physiopathology, Faculty of Pharmacy, “Victor Babeș” University of Medicine and Pharmacy, 2 Eftimie Murgu Square, 300041 Timisoara, Romania; 12 Discipline of General and Inorganic Chemistry, Faculty of Pharmacy, “Victor Babeș” University of Medicine and Pharmacy, 2 Eftimie Murgu Square, 300041 Timișoara, Romania; pop.raluca@umft.ro

**Keywords:** *Coffea arabica*, green coffee seeds, polyphenols, chlorogenic acids, antioxidant activity, antimicrobial activity, mitochondrial OXPHOS Complex I, antiproliferative activity, chorioallantoic membrane assay

## Abstract

Green seeds of *Coffea arabica* represent a rich source of bioactive compounds. This study aimed to compare the butanol-soluble (CA-BU) and the ethyl acetate-soluble (CA-EtAc) fractions in terms of their phytochemical composition and biological activity. As a first step, the fractions were analyzed by Fourier-transform infrared spectroscopy (FT-IR) and high-performance liquid chromatography coupled with mass spectrometry (HPLC–MS) in order to investigate the major constituents. Subsequently, CA-BU and CA-EtAc were evaluated for antioxidant effect, antimicrobial activity, antiproliferative properties, effects on the mitochondrial function, and on the chorioallantoic membrane. The CA-EtAc fraction was enriched in chlorogenic acids and catechins and showed superior antioxidant activity, whereas CA-BU displayed a broader profile of semi-polar polyphenols, conferring moderate antimicrobial effects and stronger antiproliferative activity in MCF-7 human breast adenocarcinoma cells, although with limited selectivity compared with HaCaT non-tumorigenic cells. Respirometric analysis demonstrated that CA-BU selectively inhibited mitochondrial oxidative phosphorylation Complex I (OXPHOS CI), without affecting Complex II (CII) or basal respiration, indicating a specific mitochondria-targeted mechanism. Both fractions were non-irritant and well tolerated in the chorioallantoic membrane (CAM) assay; CA-BU reduced vascular density. These findings demonstrate a clear mechanistic differentiation between the fractions, highlighting the decisive role of solvent polarity in obtaining extracts with distinct and targeted biological effects.

## 1. Introduction

In recent decades, plant extracts rich in bioactive compounds have attracted attention as complementary approaches for the prevention and treatment of chronic diseases, including cancer. Among natural sources, coffee extracts, especially those obtained from green *Coffea arabica* seeds, have been extensively investigated due to their high content in phenolic acids, mainly chlorogenic acids, as well as in flavonoids and diterpenes such as cafestol and kahweol [1]. These compounds exhibit a wide range of biological activities, including antioxidant, anti-inflammatory, and antitumor effects, which are essential in the fight against cancer [2,3].

Experimental data indicate that coffee extracts and their bioactive constituents can inhibit tumor cell proliferation, induce apoptosis, and modulate signaling pathways involved in carcinogenesis, inflammation, and angiogenesis [2,3]. In vitro studies have demonstrated cytotoxic effects against several cancer cell lines, including colorectal adenocarcinoma [4], oral squamous carcinoma [5], lung, esophageal, and bladder cancer [6], as well as breast cancer cells [7], suggesting antineoplastic potential.

In the context of breast cancer, chlorogenic acid has emerged as a key bioactive compound underlying these effects. [8] showed that chlorogenic acid inhibits epithelial–mesenchymal transition (EMT) and reduces invasive behavior in MCF-7 cells, effects that were also confirmed in vivo by decreased tumor growth [8]. Similarly, Hsu et al. (2021) reported that chlorogenic acid, in combination with hesperidin, exerts a synergistic inhibitory effect on MCF-7 cell proliferation, involving modulation of estrogen receptor signaling and mitochondrial function [9].

The relevance of adjuvant strategies is also supported by recent epidemiological evidence. According to the analysis, breast cancer mortality decreased by 44% between 1989 and 2022, due to early detection and standard therapies. However, breast cancer incidence continued to increase by approximately 1% annually between 2012 and 2021, particularly in hormone receptor-positive (HR+) tumors, highlighting the need for complementary approaches for long-term control [10].

Green *Coffea arabica* seeds form a complex biochemical matrix, with 59–61% carbohydrates, 11–17% lipids, 10–16% proteins, 6–10% polyphenols (predominantly chlorogenic acid (CGA)), 4% minerals, 2% fatty acids, and alkaloids such as caffeine (1–2%) and trigonelline (approximately. 1%) [11]. The phenolic and lipid fractions confer antioxidant, anti-inflammatory, and photoprotective properties due to CGA and diterpenes (cafestol, kahweol), which neutralize free radicals and modulate pro-inflammatory responses [12,13,14]. Polyphenols absorb UV radiation and protect biomolecules from oxidative stress, while roasting induces thermal degradation of polyphenols and proteins, significantly diminishing these bioactive activities [15,16].

Chlorogenic acid (CGA), present at 4.2–4.3% of the dry weight of the seeds, is the main active phenolic compound [17]. CGA, by modulating cellular metabolism and mitochondrial function, inhibits tumor cell proliferation, induces apoptosis, and suppresses metastasis by regulating NADH-dependent enzymes such as ACAT1 [18].

Due to their antioxidant and metabolism-modulating properties, CGA and CGA-rich extracts from *Coffea arabica* have been investigated for their biological potential, including in contexts relevant to oncology research. However, most existing studies focus on crude extracts, while data on fractionated extracts and their mechanisms of action remain limited. Recent studies on medicinal plant extracts have emphasized the importance of combining advanced chromatographic–mass spectrometric characterization with biological assays to establish reliable correlations between polyphenol-rich phytochemical profiles and antioxidant or anti-inflammatory activity. For example, UHPLC–high-resolution mass spectrometry-based profiling has been successfully used to identify phenolic acids and flavonoids in plant extracts and to relate these constituents to radical scavenging and other bioactivities. This analytical–biological correlation provides a relevant benchmark for studies aiming to connect solvent-dependent phytochemical composition with functional effects [19].

In this context, the present study aimed to investigate two fractions with medium polarity (ethyl acetate and butanol fractions) obtained from the crude ethanol extract of *Coffea arabica* green seeds. Such fractions are generally known to contain high amounts of phenolic compounds [20]. The sub-extracts were characterized phytochemically and evaluated biologically by correlating the chemical profiles with their effects on MCF-7 and HaCaT cell lines to assess selectivity and biological relevance.

Solvent polarity is recognized as an important factor that influences not only the extraction yield and total polyphenol content of *Coffea arabica* green seeds, but also the phytochemical profile of the resulting fractions, with potential implications for their associated biological activities. In this context, investigating the relationship between chemical composition and biological activity may provide relevant insights into the antiproliferative potential and mechanisms of action of these fractions.

The objective of this study was to characterize the phytochemical and functional properties of two *Coffea arabica* sub-extracts containing compounds with medium polarity (ethyl acetate and *n*-butanol soluble fractions, respectively). These fractions were separated from the crude ethanol extract of green coffee seeds. Moreover, we aimed to evaluate the relationship between the chemical composition and bioactivities of the two fractions, thus contributing to the rational basis for obtaining natural products with targeted biological activities.

## 2. Results

In order to evaluate the biological potential of the CA-BU and CA-EtAc fractions obtained from green seeds of *Coffea arabica*, a comprehensive phytochemical characterization was performed, followed by an in vitro assessment of their biological activities. The chemical profiles of the fractions were initially determined by evaluating the total polyphenol content (TPC) using the Folin–Ciocalteu method. The presence of functional groups characteristic of polyphenols was confirmed by Fourier-transform infrared spectroscopy (FT-IR). Individual bioactive compounds were subsequently identified and quantified by high-performance liquid chromatography coupled with mass spectrometry (HPLC–MS), enabling detailed polyphenol profiling. The fractions were then evaluated biologically by correlating their chemical profiles with effects on MCF-7 (human breast cancer) and HaCaT (human keratinocyte) cell lines to assess selectivity and biological relevance. Antioxidant and antimicrobial activities were measured to highlight the relationship between phytochemical composition and functional effects. In addition, in vivo irritation and angiogenesis were assessed using chorioallantoic membrane (CAM) models to confirm the safety of the fractions. This integrated approach allows a clear understanding of how solvent polarity impacts phytochemical composition and divergent biological activities of plant extracts, providing insight into potential mechanisms underlying selective antioxidant, antimicrobial, and mitochondria-targeted effects.

### 2.1. Extraction Yield and Polyphenol Screening

The extraction yields of the butanolic (CA-BU) and ethyl acetate (CA-EtAc) fractions, calculated relative to the dry crude ethanolic extract subjected to liquid–liquid fractionation, were 19.35% and 8.60%, respectively. These values correspond to fraction yields based on the 25.00 g of crude extract used for fractionation, rather than directly on the initial dry plant material. Both fractions showed relevant recovery, with the choice of solvent primarily affecting the specific composition of the polyphenols extracted. When expressed relative to the initial dry seed material, these values correspond approximately to 1.61% for CA-BU and 0.72% for CA-EtAc.

#### 2.1.1. Total Polyphenol Content (TPC)

The total polyphenol content (TPC), expressed in mg CAE/g extract, differed significantly between fractions. The CA-EtAc extract showed the highest value (594.89 mg CAE/g) compared to CA-BU (212.63 mg CAE/g), indicating a superior ability of ethyl acetate to extract phenolic compounds.

#### 2.1.2. Polyphenol Screening by Fourier-Transform Infrared Spectroscopy (FT-IR)

**Figure 1:**
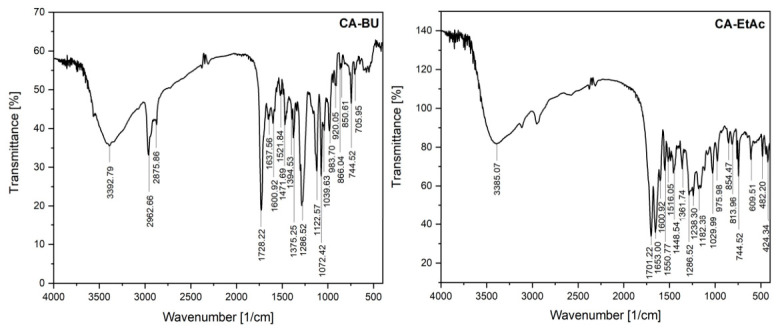
FT-IR spectra of butanolic (CA-BU) and ethyl acetate (CA-EtAc) *Coffea arabica* extracts.

The profile of functional groups of organic compounds present in CA-BU and CA-EtAc sub-extracts obtained from green seeds of *Coffea arabica* was investigated by FT-IR spectroscopy to characterize the qualitative composition of the extractive fractions (Figure 1). The main absorption bands and their tentative assignments are summarized in Table 1 and were used to compare the functional group profiles of the two fractions. Particular attention was given to the diagnostic regions corresponding to hydroxyl groups, carbonyl groups, aromatic C=C vibrations, and C–O stretching vibrations, as these signals are commonly associated with phenolic acids, flavonoids, esters, alcohols, and other oxygenated phytoconstituents.

As shown in Table 1, the FT-IR spectrum of the CA-BU fraction was characterized by a broad and intense band at 3392.79 cm^−1^, attributed to O–H stretching vibrations, characteristic of alcohols and phenolic compounds involved in intermolecular hydrogen bonding. The presence of an intense band at 1728.22 cm^−1^ indicates C=O stretching vibration, associated with carbonyl groups in esters, aldehydes, or anhydrides, suggesting the existence of oxygenated carbonyl compounds in the butanolic fraction. The bands recorded at 1286.52 cm^−1^, 1122.57 cm^−1^ and 1072.42 cm^−1^ correspond to C–O stretching vibrations, specific to aromatic esters, primary and secondary alcohols, as well as possibly aliphatic ethers. Their presence confirms the predominance of moderately polar oxygenated compounds. The signals in the 1637.56 cm^−1^ and 1600.92 cm^−1^ region are attributed to C=C stretching vibrations in alkenes (conjugated or unconjugated) and/or aromatic nuclei. This attribution is supported by the band at 1521.84 cm^−1^ (aromatic C=C) and the characteristic C–H bending band of monosubstituted aromatic compounds at 744.52 cm^−1^. The presence of aliphatic structures is indicated by the bands at 2962.66 cm^−1^ and 2875.86 cm^−1^, corresponding to C–H (–CH_3_/–CH_2_–), as well as the bands at 1471.69 cm^−1^ and 1375.25 cm^−1^, attributed to C–H bending vibrations in alkanes. The band at 1375.25 cm^−1^ may overlap contributions from phenolic O–H bending vibrations. Phenolic groupings are further suggested by the band at 1394.53 cm^−1^ (O–H bending). The possible presence of carboxylic acids is supported by both the broad O–H band and the signal at 1039.63 cm^−1^ (C–O stretching), although this region may also include contributions from primary alcohols. The bands recorded at 920.05 cm^−1^, 866.04 cm^−1^, 850.61 cm^−1^, and 705.95 cm^−1^ are attributed to C–H and C–C bending vibrations in alkenes, including cis-disubstituted configurations, confirming the presence of unsaturated structures. Overall, the FT-IR profile of the CA-BU fraction highlights a fraction rich in phenolic compounds, alcohols, esters, and unsaturated aromatic and aliphatic structures, consistent with the extraction of polar and semipolar compounds in butanol. Taken together, the bands at 3392.79 cm^−1^, 1728.22 cm^−1^, 1286.52–1072.42 cm^−1^, and 1637.56–1521.84 cm^−1^ indicate that the CA-BU fraction contains hydroxylated, carbonyl-containing, aromatic, and moderately polar oxygenated compounds, supporting the phytochemical selectivity of the butanolic fraction.

In the CA-EtAc fraction, the FT-IR peaks listed in Table 1 showed a different profile, dominated by the intense bands at 1701.22 cm^−1^ and 1653.00 cm^−1^, attributed to C=O stretching vibrations of carboxylic acids/conjugated esters and C=C stretching vibrations of conjugated alkenes, respectively. These signals indicate a fraction rich in carbonyl compounds and conjugated π systems. The band at 744.52 cm^−1^ confirms the presence of monosubstituted aromatic compounds (C–H bending out of plane), while the bands at 1550.77 cm^−1^ and 1516.05 cm^−1^ are attributed to C=C stretching vibrations of the aromatic nucleus. Phenolic and/or alcoholic groups are supported by the broad band at 3385.07 cm^−1^ (O–H stretching) and the signal at 1361.74 cm^−1^ (O–H bending). The C–O stretching vibrations recorded at 1238.30 cm^−1^, 1182.36 cm^−1^, and 1029.99 cm^−1^ indicate the presence of alcohols, but may also reflect contributions from esters, ethers, or carboxylic acids. The weak band at 1286.52 cm^−1^ suggests aromatic esters (C–O stretching). The signals at 1448.54 cm^−1^, 975.98 cm^−1^, 854.47 cm^−1^, and 813.96 cm^−1^ are attributed to C–H and C–C bending vibrations in alkenes, including cis or trisubstituted configurations. A weak signal at 609.51 cm^−1^ may be associated with C–H bending vibrations in alkynes. Both fractions show spectral signatures characteristic of phenolic, aromatic, and carbonyl compounds, but the CA-BU fraction shows a more pronounced presence of hydroxyl groups and aliphatic structures, while the CA-EtAc fraction is dominated by conjugated carbonyl systems and aromatic structures [21]. The differences observed confirm the influence of solvent polarity on the phytochemical profile of the fractions obtained. Therefore, the cross-referenced FT-IR peaks in Table 1 indicate that the CA-EtAc fraction is comparatively enriched in conjugated carbonyl and aromatic systems, whereas the CA-BU fraction shows stronger contributions from hydroxylated and aliphatic structures. This spectral differentiation supports the role of solvent polarity in shaping the chemical profile of the two fractions.

#### 2.1.3. HPLC Analysis of Polyphenols

The polyphenolic composition of *Coffea arabica* green seed fractions, namely the butanolic fraction (CA-BU) and the ethyl acetate fraction (CA-EtAc), was analyzed by high-performance liquid chromatography coupled with mass spectrometry (HPLC–MS), and the results are summarized in Table 2. The compounds reported in Table 2 were selected as relevant phenolic markers for the two fractions, allowing a direct comparison between chlorogenic acid derivatives and catechin-type compounds. This cross-reference was used to interpret the solvent-dependent distribution of phenolic acids and flavan-3-ols in CA-BU and CA-EtAc fractions. For each compound, a limit of detection (LOD) of 0.1 µg/mL was established, defined as the minimum concentration producing a reproducible peak with a signal-to-noise ratio > 3 [21].

Both fractions showed high concentrations of chlorogenic acid (>48 μg/mL), with 4-O-caffeoylquinic acid reaching the highest level in CA-BU (7.931 μg/mL). Catechins (epicatechin, epigallocatechin, and epigallocatechin gallate) were detected only in the CA-EtAc fraction (0.134 ± 0.012, 0.995 ± 0.069, and 1.68 ± 0.252 µg/mL, respectively), under the applied HPLC–MS conditions, while these compounds were not detected in CA-BU. This solvent-guided enrichment in catechin-type compounds also supports the superior antioxidant activity of the CA-EtAc fraction, since flavan-3-ols are efficient hydrogen- and electron-donating antioxidants.

The HPLC–MS data summarized in Table 2 indicate both similarities and clear differences between the two fractions. Chlorogenic acid was the major quantified phenolic compound in both CA-BU and CA-EtAc fractions, with comparable concentrations of 50.971 ± 2.038 µg/mL and 48.554 ± 0.485 µg/mL, respectively. In contrast, 4-O-caffeoylquinic acid was more abundant in the CA-BU fraction (7.931 ± 0.396 µg/mL) than in the CA-EtAc fraction (2.994 ± 0.209 µg/mL), suggesting preferential partitioning of this caffeoylquinic acid derivative into the butanolic fraction. Conversely, catechin-type compounds were detected only in CA-EtAc, including epicatechin, epigallocatechin, and epigallocatechin gallate. This distribution indicates that ethyl acetate selectively enriched the fraction in flavan-3-ols, while butanol favored a profile dominated by chlorogenic acid derivatives. This pattern suggests a solvent-dependent partitioning of flavan-3-ols during sequential liquid–liquid fractionation, rather than a uniform distribution of all phenolic subclasses between the two medium-polarity fractions. These differences provide a phytochemical basis for the divergent biological activities observed between the two fractions. In particular, the CA-BU profile was mainly characterized by chlorogenic acid and a comparatively higher amount of 4-O-caffeoylquinic acid, identifying these caffeoylquinic acid derivatives as the main HPLC–MS-detected candidates potentially associated with the mitochondrial effects observed for this fraction.

### 2.2. Antioxidant Activity

**Figure 2:**
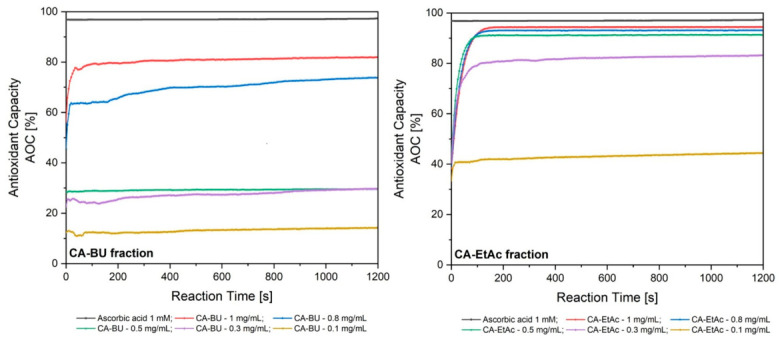
Time-dependent antioxidant capacity (AOC%) of the butanolic (CA-BU) and ethyl acetate (CA-EtAc) fractions obtained from green *Coffea arabica* seeds, evaluated by the DPPH radical scavenging assay at concentrations ranging from 0.1 to 1 mg/mL. Ascorbic acid 1 mM was used as a positive control. Data are expressed as mean ± SD of three independent determinations (*n* = 3); SD values are reported in Table 3.

The antioxidant capacity of the CA-BU and CA-EtAc fractions from green *Coffea arabica* seeds was evaluated using the 2,2-diphenyl-1-picrylhydrazyl (DPPH) radical scavenging assay at five concentrations (0.1–1 mg/mL) and compared with 1 mM ascorbic acid (Table 3, Figure 2). The results were expressed as antioxidant capacity (AOC, %) and half-maximal effective concentration (EC_50_).

The DPPH test results showed that the CA-EtAc extract has a clearly superior antioxidant activity compared to the CA-BU extract. At the first three concentrations tested (1, 0.8, and 0.5 mg/mL), CA-EtAc recorded inhibitions of 94%, 93%, and 91%, approaching the standard of 1 mM ascorbic acid (97%). At 0.3 mg/mL, the inhibition was approximately 80%, and at 0.1 mg/mL, approximately 40%. Except for the lowest concentration, DPPH radical consumption occurred within the first 200 s, rapidly reaching kinetic equilibrium; at 0.1 mg/mL, maximum radical consumption occurred in approximately 80 s.

In contrast, the CA-BU extract showed lower antioxidant activity: the maximum concentration tested (1 mg/mL) was equivalent to 0.3 mg/mL for the CA-EtAc extract, and the other concentrations required a longer time for radical consumption. At 0.8 mg/mL, complete consumption of DPPH radicals was not achieved in 20 min, and at 0.1 mg/mL, activity remained >10%. No significant differences were observed between 0.5 mg/mL and 0.3 mg/mL, especially at the end of the analysis period.

Both fractions showed concentration-dependent antioxidant activity, correlated with their polyphenol content, confirming the superior efficacy of the ethyl acetate fraction.

The EC_50_ was calculated by linear regression analysis, taking into account the AOC values obtained and their concentrations. The EC_50_ of *Coffea arabica* butanolic fraction was 0.55 ± 0.08 mg/mL (R^2^ = 0.8692), whereas the EC_50_ of *Coffea arabica* ethyl acetate fraction was 0.11 ± 0.005 mg/mL (R^2^ = 0.9930).

### 2.3. Biological Assessment

#### 2.3.1. Antimicrobial Activity

The antimicrobial activity of the *Coffea arabica* CA-BU and CA-EtAc fractions was evaluated using the disk diffusion method (DDM). The inhibition zone diameters are presented in Table 4.

The results demonstrated strain-dependent antimicrobial effects. The butanolic fraction (CA-BU) exhibited moderate antibacterial activity against *Staphylococcus aureus* and *Escherichia coli*, with inhibition zone diameters of 12 mm for both strains. Reduced activity was observed against *Pseudomonas aeruginosa*, indicating lower susceptibility among Gram-negative bacteria compared to Gram-positive strains.

The ethyl acetate fraction (CA-EtAc) showed overall lower antibacterial activity than the butanolic fraction (CA-BU). However, both fractions (CA-BU and CA-EtAc) displayed weak antifungal activity against *Candida parapsilosis*, with inhibition zones of approximately 11 mm.

According to the applied interpretative criteria, inhibition zones ranging from 6 to 15 mm indicate low susceptibility. Therefore, the DDM results should be interpreted as a preliminary qualitative screening rather than as evidence of strong antimicrobial activity. Since the inhibition zones were small, ranging from 7 to 12 mm, minimum inhibitory concentration (MIC) determination was not performed at this stage. Nevertheless, the CA-BU fraction showed comparatively higher inhibition zones than the CA-EtAc fraction, particularly against *Staphylococcus aureus* and *Escherichia coli*, suggesting that this fraction may warrant further quantitative antimicrobial evaluation by broth microdilution.

#### 2.3.2. Antitumor Activity

- Cytotoxicity Evaluation

**Figure 3:**
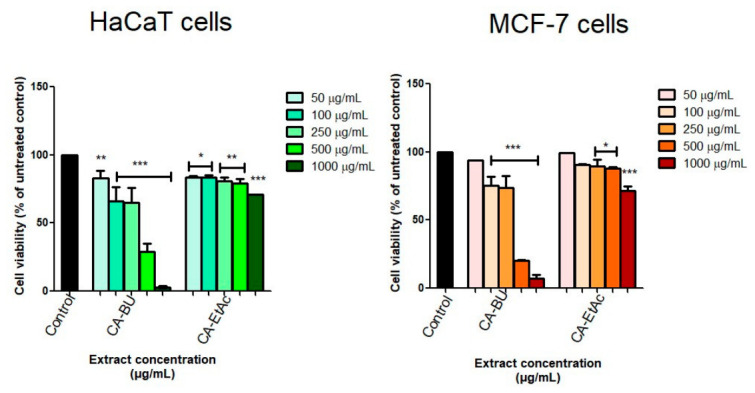
Viability of HaCaT keratinocytes and MCF-7 breast adenocarcinoma cells after 48 h of treatment with the butanolic (CA-BU) and ethyl acetate (CA-EtAc) fractions obtained from green *Coffea arabica* seeds at concentrations of 50, 100, 250, 500, and 1000 μg/mL. Cell viability was assessed using the Alamar Blue assay and expressed as a percentage of the untreated control, considered 100%. Data are expressed as mean ± SD of three independent experiments, each performed in triplicate (*n* = 3). Statistical significance was calculated versus untreated control cells using one-way ANOVA followed by Dunnett’s post hoc test: * *p* < 0.05; ** *p* < 0.01, and *** *p* < 0.001.

The cytotoxic potential of the butanol (CA–BU) and ethyl acetate (CA–EtAc) fractions was evaluated in HaCaT keratinocytes and MCF-7 breast adenocarcinoma cells after 48 h of treatment (50, 100, 250, 500, 1000 μg/mL) using the Alamar Blue assay. Statistical significance was assessed versus untreated control cells, and significant differences are indicated directly in Figure 3 using standardized annotations.

In HaCaT cells, CA–EtAc showed low cytotoxicity, maintaining viability above 70% at 500 μg/mL (79.26% ± 3.38) and 1000 μg/mL (70.83% ± 0.02), whereas CA–BU significantly reduced viability even at lower concentrations, with a half-maximal inhibitory concentration (IC_50_) of 369.4 ± 1.02 μg/mL.

In MCF-7 cells, CA–BU was the most potent, decreasing viability at 100–250 μg/mL (IC_50_ = 312.24 ± 0.51 μg/mL), while CA–EtAc only exhibited moderate inhibition at 500 μg/mL (79.26% ± 3.38) and 1000 μg/mL (70.83% ± 0.02) (Figure 3). The selectivity index of the CA-BU fraction, calculated as the ratio between the IC_50_ in HaCaT cells and the IC_50_ in MCF-7 cells, was approximately 1.18, indicating a narrow in vitro therapeutic window.

These results indicate a stronger antiproliferative effect of CA–BU in MCF-7 cells, with limited cytotoxicity of CA–EtAc in non-tumorigenic keratinocytes.

- The effect of CA-BU in high-resolution respirometry studies

**Figure 4:**
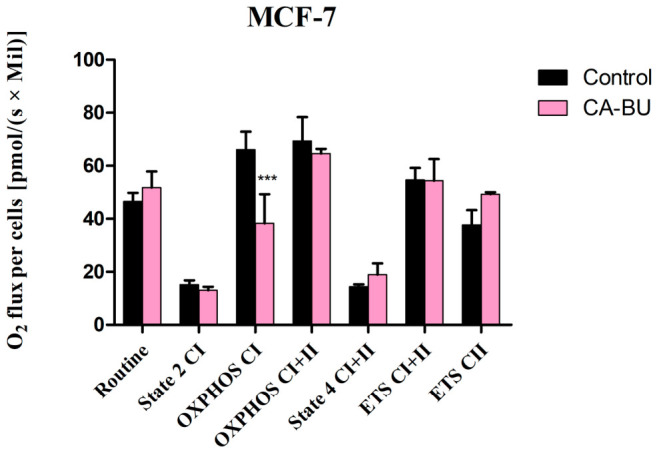
Mitochondrial respiration of permeabilized MCF-7 breast adenocarcinoma cells after treatment with the butanolic fraction CA-BU at its IC_50_ concentration (312.24 ± 0.51 μg/mL). Data are expressed as mean ± SD of four independent experiments (*n* = 4). Statistical significance was calculated versus untreated control cells using one-way ANOVA followed by Dunnett’s post hoc test: * *p* < 0.05, ** *p* < 0.01, and *** *p* < 0.001.

Several studies have analyzed the role of mitochondria in inducing apoptosis in mammalian cells [22]. To determine whether the anticancer effects of CA-BU against MCF-7 cells could be associated with mitochondrial function, CA-BU was tested at its IC_50_ value of 312.24 ± 0.51 μg/mL on permeabilized MCF-7 cells using high-resolution respirometry (Figure 4). The analysis focused on oxidative phosphorylation (OXPHOS), mitochondrial Complex I (CI), mitochondrial Complex II (CII), and the electron transfer system (ETS) respiratory states. The values recorded revealed that CA-BU selectively inhibited active respiration, namely OXPHOS Complex I (38.26 ± 4.38), compared to the control group (66.25 ± 5.8), suggesting that the compound might act as a NADH oxidation modulator, without affecting the succinate oxidation, OXPHOS Complex II (64.63 ± 1.24 vs. 69.45 ± 7.81). Moreover, CA-BU did not significantly affect basal respiration routine (51.82 ± 6.16 vs. 46.64 ± 2.57), State 2_CI_ (13.03 ± 0.48 vs. 15.27 ± 1.35) and State 4_CI+CII_ (19.01 ± 4.25 vs. 14.54 ± 0.71) or the maximal respiration in the uncoupled state—ETS (ETS_CI+CII_: 54.37 ± 8.11 vs. 54.76 ± 3.67 and ETS_CI_: 49.17 ± 0.5 vs. 37.81 ± 4.84) in a significant manner.

#### 2.3.3. In Vivo Evaluation of Irritation and Angiogenesis

- Hen’s Egg Test on the Chorioallantoic Membrane (HET-CAM) Irritation Assessment

**Figure 5:**
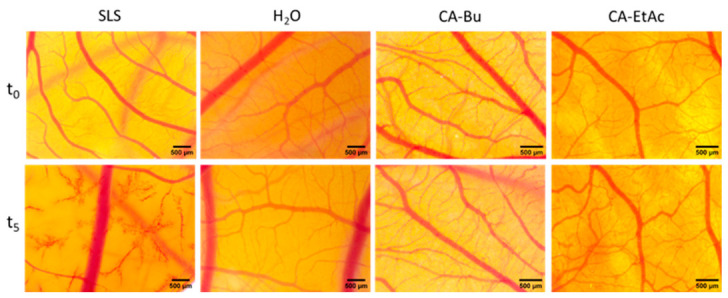
Representative stereomicroscopic images showing the effects induced by the butanolic (CA-BU) and ethyl acetate (CA-EtAc) fractions obtained from green *Coffea arabica* seeds on the chorioallantoic membrane (CAM) using the HET-CAM irritation assay. The images document the absence of hemorrhage, vascular lysis, and coagulation after application of the tested fractions. Experiments were performed in triplicate (*n* = 3). Scale bars represent 500 µm.

At 400 µg/mL, both CA-BU and CA-EtAc fractions were classified as non-irritant in the Hen’s Egg Test on the Chorioallantoic Membrane (HET-CAM), according to the Luepke scale [23]. No hemorrhage, vascular lysis, or coagulation events were observed during the 300 s monitoring period, and the calculated irritation scores were comparable to those of the negative (H_2_O) (Table 5). In contrast, sodium lauryl sulphate (SLS) induced marked vascular damage and was categorized as a strong irritant (Figure 5). These findings indicate good mucosal and epithelial tolerability of both fractions at a biologically active concentration.

- Angiogenesis Evaluation

**Figure 6:**
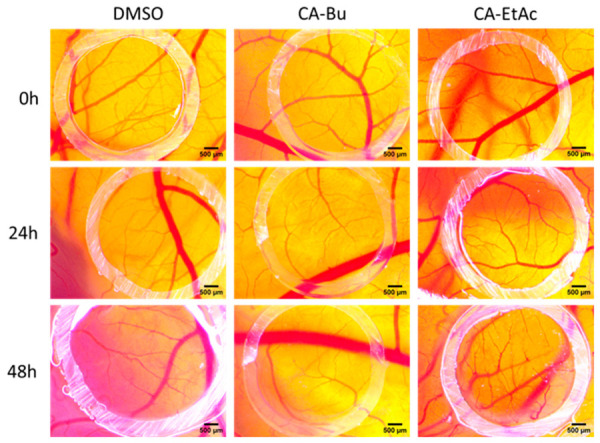
Representative stereomicroscopic images illustrating the effects of the butanolic (CA-BU) and ethyl acetate (CA-EtAc) fractions obtained from green *Coffea arabica* seeds on CAM angiogenesis at 0, 24, 48, and 72 h post-treatment. DMSO was used as a solvent control. Experiments were performed in triplicate (*n* = 3). Scale bars represent 500 µm.

In the angiogenesis assay, daily stereomicroscopic monitoring over 48 h revealed no disruptive vascular reactions following treatment with either fraction. CA-EtAc did not induce noticeable changes in vessel density or branching pattern compared to the control. In contrast, CA-BU treatment resulted in a slight reduction in vascular density after 48 h, without signs of vascular regression or toxicity (Figure 6). The absence of vascular damage and avascular zones, together with the slight decrease in vascular density observed for CA-BU, indicates a mild attenuation of ongoing angiogenic processes, consistent with a modulatory rather than an anti-angiogenic response.

## 3. Discussion

The results confirm that CA–EtAc and CA–BU fractions from green *Coffea arabica* seeds are dominated by chlorogenic acids, consistent with other references on semipolar green coffee extracts [12,13,14,24,25]. Chlorogenic acid and 4-O-caffeoylquinic acid play a central role in antioxidant activity through hydrogen donation and electron transfer [12,24].

Quantitative differences between fractions reflect solvent selectivity and confirm semipolar solvent efficiency for specific polyphenol extraction [13,14,24,25]. LC–MS/MS allowed reproducible phenolic profiling and direct chemical–biological activity correlation [14].

FT-IR data confirmed typical phenolic functional groups, consistent with previous reports, and demonstrated solvent-driven phytochemical selectivity [26,27,28]. The cross-referenced FT-IR assignments in Table 1 further support this interpretation, as CA-BU displayed prominent hydroxyl, C–O, aliphatic C–H, and aromatic signals, whereas CA-EtAc showed stronger contributions from conjugated carbonyl and aromatic C=C bands. These spectral differences are consistent with the HPLC–MS profile shown in Table 2, where both fractions contained chlorogenic acid, but CA-EtAc was additionally characterized by catechin-type compounds.

TPC is influenced by environmental and extraction parameters [15,16]. The higher TPC in CA-EtAc confirms the efficiency of ethyl acetate for medium-polar phenolics.

Antioxidant activity results are consistent with chlorogenic acid and flavonoid dominance [29,30]. CA-EtAc showed superior DPPH scavenging activity [21,30,31]. CA-BU activity at higher concentrations likely reflects phytochemical interactions [7,30,32,33].

HPLC–MS analysis revealed a clear segregation of bioactive compounds between the fractions, providing a mechanistic explanation for the observed functional differences. The high content of chlorogenic acids and catechins in the CA-EtAc fraction correlates directly with its superior antioxidant capacity, as these compounds are well known for efficiently neutralizing free radicals through electron and hydrogen transfer mechanisms. The exclusive detection of catechin-type compounds in the CA-EtAc fraction can be explained by solvent-dependent partitioning during sequential liquid–liquid fractionation. Although both ethyl acetate and *n*-butanol are considered medium-polarity solvents, their extraction behavior differs markedly. Ethyl acetate is an intermediate-polarity, aprotic solvent that can efficiently solubilize phenolic compounds with aromatic structures and moderate polarity, including flavan-3-ols such as epicatechin, epigallocatechin, and epigallocatechin gallate. In contrast, *n*-butanol is more polar and protic, with stronger hydrogen-bonding capacity, which may favor the partitioning of more hydrophilic hydroxylated compounds, such as caffeoylquinic acid derivatives, rather than catechin-type flavan-3-ols. Moreover, the sequential extraction scheme may have depleted catechins into the ethyl acetate phase before the *n*-butanol step, explaining why these compounds were below the detection limit in the CA-BU fraction. Therefore, the absence of detectable catechins in the CA-BU fraction should be interpreted as a consequence of preferential partitioning and detection limits, rather than as definitive evidence that these compounds were completely absent from the initial crude extract. In contrast, the CA-BU fraction, characterized by a broader spectrum of semi-polar and hydroxylated compounds but lacking detectable catechins, exhibited stronger antimicrobial and antiproliferative activities. This profile suggests that the biological effects of CA-BU are predominantly mediated through cellular and mitochondrial interactions rather than direct antioxidant activity, a hypothesis supported by the selective inhibition of OXPHOS Complex I observed via high-resolution respirometry.

The antimicrobial assay should be interpreted as a preliminary screening rather than as a definitive quantitative assessment. The inhibition zones obtained for both fractions ranged from 7 to 12 mm, indicating low microbial susceptibility according to the applied criteria. Although the CA-BU fraction produced comparatively larger inhibition zones against *Staphylococcus aureus* and *Escherichia coli*, these values do not support strong antimicrobial activity. Therefore, the observed effects should be regarded as modest and exploratory. MIC determination by broth microdilution would be required in future studies to quantify antimicrobial potency, establish concentration-dependent effects, and enable direct comparison with other plant-derived extracts and antimicrobial standards. The low susceptibility observed here is consistent with previous reports on crude or partially purified phenolic extracts, where the antimicrobial response may be influenced by compound diffusion, extract complexity, and intrinsic microbial resistance mechanisms [34,35,36,37].

The results of this study reveal clear differences between CA–BU and CA–EtAc fractions in terms of their cytotoxic effects on HaCaT keratinocytes and MCF-7 breast adenocarcinoma cells, highlighting the importance of phytochemical composition and cellular context. The CA–EtAc fraction showed low cytotoxicity, maintaining HaCaT viability above 70% even at high concentrations (500–1000 μg/mL) and inducing a modest reduction in MCF-7 viability at the upper concentration range. This profile is consistent with the literature describing the semipolar fractions of *Coffea arabica* as being relatively well tolerated by non-tumorigenic cells and characterized by limited antiproliferative activity when used alone [38].

In contrast, the butanolic fraction (CA–BU) induced a pronounced reduction in cell viability in both cell lines, with MCF-7 cells being more susceptible (IC_50_ = 312.24 ± 0.51 μg/mL) than HaCaT cells (IC_50_ = 369.4 ± 1.02 μg/mL). However, the calculated selectivity index, defined as IC_50_ HaCaT/IC50 MCF-7, was approximately 1.18, indicating only limited selectivity toward tumor cells. This narrow in vitro therapeutic window has important translational implications. Although the CA-BU fraction displays relevant antiproliferative activity and a mitochondria-associated mechanism in MCF-7 cells, the comparable cytotoxicity observed in non-tumorigenic HaCaT keratinocytes suggests that the fraction, in its current non-purified form, cannot be considered selectively antitumoral. Therefore, the present findings should be interpreted primarily as mechanistic and phytochemical evidence rather than as direct proof of clinical applicability. Further purification, identification of the active constituents, optimization of the fraction composition, and evaluation in additional non-tumorigenic and tumor cell models are required to improve selectivity and define a safer therapeutic window. The findings partially align with studies reporting antiproliferative effects of coffee extracts and chlorogenic acid (CGA) on MCF-7 cells, but also challenge the frequently implied ER+ selectivity, which is often reported without direct comparison to non-tumorigenic cells [9,39].

Hsu et al. demonstrated that chlorogenic acid (CGA) inhibits the proliferation of MCF-7 cells in a dose-dependent manner. The IC_50_ value, determined by the MTT assay after 72 h of treatment, was approximately 350 μM, indicating moderate cytotoxicity [9].

Furthermore, the molecular mechanisms proposed in the literature remain fragmented and, at times, contradictory. Hsu et al. describe an estrogen receptor-dependent mitochondrial mechanism independent of reactive oxygen species [9]. Wang et al. report, in co-treatment contexts, mitochondrial dysfunction associated with oxidative stress, suggesting a contextual mechanism of action dependent on the combination of compounds and experimental conditions [40]. Li et al. demonstrated that puerarin-loaded mitochondria-targeted micelles significantly enhanced mitochondrial drug accumulation while reducing lysosomal sequestration compared with free puerarin, indicating that nanoparticle-based incorporation may improve mitochondria-directed therapeutic outcomes [41]. Given the pronounced cytotoxicity of the CA–BU fraction on MCF-7 cells and inconsistent data in the literature regarding the involvement of mitochondria in the action of polyphenols in *Coffea arabica*, direct investigation of mitochondrial function was warranted.

Apoptosis mainly occurs through 2 distinct pathways, namely the extrinsic pathway, mediated by different receptors, and the intrinsic pathway that is mitochondrial-mediated [42].

To assess whether the apoptotic mechanism was mitochondria-mediated, we performed high-resolution respirometry studies of the fraction obtained with the highest cytotoxic activity (CA-BU) in MCF-7 breast adenocarcinoma cells. The results revealed that this fraction inhibited active respiration, more specifically OXPHOS_CI_, but did not alter OXPHOS_CI+CII_ or the electron transport chain maximal respiratory capacity (ETS_CI_ and ETS_CI+CII_), thus suggesting the compound exerts a mild impairment of the mitochondrial function [43].

Recent literature indicates that targeting the mitochondrial electron transport chain (ETC) represents a promising anticancer strategy [44]. In particular, mitochondrial Complex I (CI) plays a key role in electron transport, oxidative phosphorylation (OXPHOS), and maintenance of the NAD^+^/NADH balance, thereby supporting cancer cell survival under metabolic stress [45]. Moreover, mitochondrial dysfunction associated with excessive reactive oxygen species (ROS) production can activate regulated cell death pathways, including pyroptosis, and contribute to tumor growth inhibition [46]. In this context, and in agreement with our respirometric data, CA-BU appears to predominantly affect CI-linked respiration, acting as a modulator of NADH oxidation without significantly altering succinate-driven CII respiration [47]. This selectivity is consistent with the fact that CI, unlike CII, directly uses NADH as an electron donor.

HPLC–MS analysis identified chlorogenic acid as the major phenolic compound in CA-BU, together with a relative enrichment of 4-O-caffeoylquinic acid compared with CA-EtAc, suggesting preferential accumulation of caffeoylquinic acid derivatives in the butanolic fraction. Such mechanistic behavior is consistent with the observed selective decrease in CI-linked OXPHOS in MCF-7 cells, without a corresponding inhibition of CII-supported respiration. Among the identified constituents, chlorogenic acid and 4-O-caffeoylquinic acid are therefore the most plausible contributors to the observed modulation of NADH oxidation, given both their relative abundance and their known redox properties. However, since individual compounds were not tested separately, a direct causal relationship cannot be established. The observed effect may instead reflect additive or synergistic interactions within the CA-BU phytocomplex, including semi-polar constituents not individually quantified by the targeted HPLC–MS approach.

Given the relatively narrow selectivity index observed in viability assays, this mitochondria-related effect should be interpreted primarily as a mechanistic indication of bioactivity rather than evidence of selective anticancer efficacy. In line with previous reports, mitochondrial targeting efficiency can be significantly enhanced by appropriate delivery systems [41].

Overall, our findings support the involvement of CI as a potential target of CA-BU-derived bioactive compounds, particularly caffeoylquinic acid derivatives, although further studies using isolated constituents and optimized delivery strategies are required to elucidate the precise molecular mechanisms and improve selectivity.

The unmodified routine state, which represents the physiological control of cellular substrate uptake, shows that the CA-BU does not influence the mitochondrial ATP demand [48]. Furthermore, CA-BU does not influence State 2_CI_ and State 4_CI+CII_, thus revealing that compound is not able to alter the non-phosphorylating resting state of decoupled or uncoupled respiration and hence it does not act like a mitochondrial uncoupler; several studies have indicated that the inhibition of the uncoupling proteins could enhance the effects of chemotherapeutic agents by strengthening ROS signals and promoting superoxide generation leading to the oxidative injury of cancer cells [49].

The HET-CAM test demonstrated that both fractions, CA-BU and CA-EtAc, are non-irritating, confirming high compatibility with epithelial and vascular tissues. This result is fully consistent with previous in vivo studies showing that ethanolic and aqueous extracts from green coffee beans, including nano- and lipid-formulations, do not induce hemorrhage, coagulation, or vascular lysis and are well tolerated by mucosal tissues [50,51]. These data validate the CAM model as a suitable tool for evaluating the biocompatibility of coffee-derived phytocomplexes.

Although both fractions were classified as non-irritating, after 48 h of exposure, the CA-BU fraction induced a slightly reduced vascular density compared to the control, suggesting a mild regulatory effect on angiogenesis rather than vascular toxicity. This observation is consistent with the known ability of chlorogenic acid derivatives to modulate endothelial proliferation [52]. The presence of flavan-3-ols in CA-EtAc may partially explain its distinct biological profile. Epigallocatechin gallate (EGCG) has been shown to induce ROS-dependent apoptosis in cancer cells by modulating redox-mediated signaling [53].

This mechanism aligns with the strong antioxidant activity and improved epithelial tolerability observed for CA-EtAc. In contrast, the absence of catechins in CA-BU may favor alternative redox-associated mechanisms underlying its enhanced tumor cell susceptibility and antimicrobial effects. In contrast, the catechin-containing CA-EtAc fraction exhibited superior radical scavenging potency (lower EC50) and better epithelial tolerability, likely due to the well-documented ROS-quenching and cytoprotective properties of flavan-3-ols such as epigallocatechin gallate (EGCG) [54]. This redox stabilizing capacity is consistent with the absence of irritative effects and vascular alterations observed in the CAM model, supporting its compatibility with mucosal tissues.

Previous studies have shown that green coffee extracts remain non-toxic and well-tolerated for several days after administration on CAM, reinforcing the observed safety profile. Furthermore, existing data indicate that chlorogenic acid-rich extracts may stimulate angiogenesis in certain physiological contexts. Water-soluble fractions from green coffee seeds demonstrated stimulation of neovascularization at 48 and 72 h, as well as after prolonged incubation periods, without affecting tissue integrity [55].

These observations are consistent with recent evidence that polyphenol-rich coffee extracts exert multifaceted anticancer actions, including suppression of tumor cell proliferation, modulation of oxidative stress, and impact on angiogenesis-related processes. Chlorogenic acids, the main phenolic constituents of *Coffea arabica*, have been implicated in preventing capillary regression under oxidative stress and modulating the balance of pro- and anti-angiogenic factors in vivo [56]. The CAM assay was performed during embryonic days 8–10, a period characterized by intense physiological angiogenesis. In this highly pro-angiogenic environment, the slight reduction in vascular density observed with CA-BU, in the absence of vascular regression or structural alterations, suggests a mild attenuation of ongoing angiogenic activity rather than a direct anti-angiogenic effect.

Moreover, comprehensive reviews indicate that coffee polyphenols and other bioactive constituents contribute to anticancer effects through mechanisms linked to redox regulation, apoptosis, and inhibition of tumor progression across multiple model systems [57]. The absence of irritative effects and the maintenance of vascular integrity in the HET-CAM test support the potential of these fractions for topical, mucosal, and regenerative applications, where high biocompatibility and angiogenesis control are essential.

Overall, the differences observed between the CA-EtAc and CA-BU fractions highlight the relevance of the extraction solvent in modulating the phytochemical profile and, consequently, the associated biological activities, with CA-EtAc showing higher antioxidant capacity and CA-BU displaying more pronounced antimicrobial, antiproliferative, and mitochondria-targeted effects. These findings emphasize the importance of polarity-driven fractionation as a key tool for tailoring phytochemical profiles to specific biological responses, paving the way for mechanism-oriented studies and targeted applications.

## 4. Materials and Methods

### 4.1. Extraction Procedure and Extraction Yield Assessment

The extraction departed from the obtainment of a crude ethanol extract. This extract was suspended in water, subjected to depletion of non-polar and low-polar compounds with petroleum ether and diethyl ether, then extracted with ethyl acetate and butanol to yield medium-polarity fractions.

**Figure 7:**
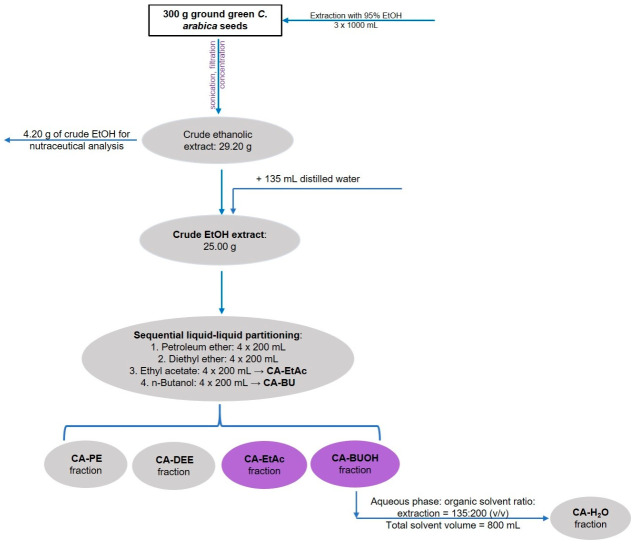
Fractionation diagram showing the preparation of *Coffea arabica* green seed fractions. Ground seeds were extracted with 95% ethanol, and 25.00 g of the dried crude ethanolic extract were suspended in 135 mL distilled water. Liquid–liquid partitioning was performed sequentially with petroleum ether, diethyl ether, ethyl acetate, and *n*-butanol, using 4 × 200 mL of each solvent. The aqueous phase-to-organic solvent ratio was 135:200 (*v*/*v*) for each extraction step. CA-EtAc, ethyl acetate fraction; CA-BU, *n*-butanol fraction.

In detail, 300 g of green *Coffea arabica* seeds were ground using an IKA A11 basic equipment (IKA-Werke GmbH & Co. KG, Staufen, Germany), and the powder obtained was mixed with 1000 mL of 95% ethanol (EtOH 95%). The mixture was left to macerate for 24 h at 22 ± 2 °C. After maceration, the mixture was sonicated for 30 min using an ultrasonic water bath (ELMA S120 Elmasonic from Elma Schmidbauer GmbH, Singen, Germany). Subsequently, filtration was performed using Whatman Grade No. 4 filter paper. The plant residue was extracted two more times with 1 L of 95% ethanol each time. The filtered extracts were united and concentrated using a rotary evaporator (Laborata 4000eco from Heidolph Instruments, GmbH & Co. KG, Schwabach, Germany) at a pressure of 97 mbar and a temperature of 35 °C. After concentration, 29.20 g of crude extract was obtained. Therefore, 25.00 g was subjected to fractionation. As a first step, the crude extract was suspended in 135 mL of distilled water, stirred, and transferred to a separating funnel. The aqueous suspension, therefore, consisted of 25.00 g of dry crude ethanolic extract dispersed in 135 mL of distilled water. Subsequently, solvents with increasing polarity, petroleum ether, diethyl ether, ethyl acetate, and *n*-butanol, were added sequentially to the aqueous suspension, as shown in Figure 7. Each liquid–liquid extraction step was performed four times using 200 mL of the corresponding solvent per extraction. Thus, for each solvent, a total volume of 800 mL was used. The aqueous phase-to-organic solvent ratio was 135:200 (*v*/*v*) for each individual extraction step, corresponding approximately to 1:1.48, and 135:800 (*v*/*v*) for the cumulative extraction with each solvent. After each extraction step, the phases (aqueous phase and organic phase) were separated, and the organic phases were concentrated. The organic phases obtained with each solvent were pooled separately and evaporated to dryness under reduced pressure. Fraction yields were calculated based on the dry mass of each recovered fraction relative to the dry mass of crude ethanolic extract subjected to fractionation, according to the following equation:

$$Yield\left(\%\right)=\frac{m_{f}}{m_{c}}\times 100$$

where $m_{f}$ is the mass of the dried recovered fraction, and $m_{c}$ is the mass of the dry crude ethanolic extract subjected to fractionation.

Therefore, the reported yields for CA-EtAc and CA-BU fractions refer to the 25.00 g of dry crude ethanolic extract used for fractionation and not directly to the initial 300 g of dry green coffee seed material. The fractions with medium polarity: ethyl acetate (CA-EtAc) and *n*-butanol (CA-BU) were further analyzed in the current research. They were stored at 4 °C in a refrigerator until further use.

### 4.2. Polyphenol Screening by TPC, FT-IR, and HPLC-MS/MS

- Determination of total phenolic content (TPC)

The total phenolic content (TPC) in the butanolic and ethyl acetate fraction obtained from green seeds of *Coffea arabica* was determined using the Folin–Ciocalteu method, with chlorogenic acid as the standard [58]. A total of 2 mg of each extract was extracted with 1 mL of a solvent mixture of acetone: methanol: distilled water: acetic acid (40:40:20:0.1, *v*/*v*/*v*/*v*) at 60 °C for 1 h, followed by sonication for 30 s. From each extract, 200 µL were analyzed in triplicate by reaction with Folin–Ciocalteu reagent and 7.5% (*m*/*v*) sodium carbonate. After incubation at room temperature for 2 h in the dark, the absorbance was measured at 726 nm (Synergy H1 Hybrid Multi-Mode Reader, Agilent BioTek, Winooski, VT, USA). The results were expressed as mg equivalents of chlorogenic acid per gram of dry material (mg EAC/g DM).

- Fourier-transformed infrared spectroscopy (FT-IR)

The organic functional groups of the phytochemical compounds in the extracts (CA-BU and CA-EtAc) were investigated through the FT-IR spectroscopy qualitative method, using the Prestige-21 spectrometer (Shimadzu, Duisburg, Germany). Each extract was mixed with KBr until a pellet was obtained and further analyzed at room temperature (22 ± 2 °C) in the spectral region from 4000 cm^−1^ to 400 cm^−1^, with a resolution of 4 cm^−1^.

The spectra were interpreted based on the match between the recorded absorption bands of the extracts of *Coffea arabica* green seeds at a specific wavenumber and the absorption band frequencies contained in the electronic library [59].

- HPLC–MS Analysis

*Chemicals and reagents*

All solvents and reagents used in this study were of analytical or HPLC grade. Analytical-grade methanol and acetic acid were purchased from Merck KGaA (Darmstadt, Germany). Ultrapure water, obtained via a Milli-Q purification system, was used throughout the analytical procedures.

For both employed analytical methods, high-purity reference standards were acquired. The following standards were purchased from Sigma-Aldrich (Sigma-Aldrich Chemie GmbH, Schnelldorf, Germany) and are listed in alphabetical order: 4-*O*-caffeoylquinic acid, apigenin, caffeic acid, caftaric acid, (+)-catechin, chlorogenic acid, *p*-coumaric acid, (–)-epicatechin, epigallocatechin (EGC), epigallocatechin gallate (EGCG), fisetin, gentisic acid, hyperoside, isoquercitrin, kaempferitrin, kaempferol, kaempferol-3-*O*-rhamnoside, luteolin, myricetin, patuletin, protocatechuic acid, quercetin, quercitrin, rutin, syringic acid, vanillic acid, vitexin, and vitexin-2-*O*-rhamnoside. Additional reference standards were purchased from other suppliers: ferulic acid and gallic acid were obtained from Merck (Darmstadt, Germany), while sinapic acid was purchased from Carl Roth (Karlsruhe, Germany).

*Analytical methods employed*

The identification and quantification of polyphenols present in butanolic and ethyl acetate fractions obtained from green seeds of *Coffea arabica* was performed by high performance liquid chromatography coupled with mass spectrometry (HPLC-MS), using two distinct previously validated analytical methods [60,61,62]. The dried fractions were analyzed after solubilization in methanol at a concentration of 1 mg/mL. The analyses were performed on an HPLC Series system coupled with a mass spectrometer (LC/MSD Ion Trap SL from Agilent1100) from Agilent Technologies (Santa Clara, CA, USA) [61].

For the first LC-MS/MS analytical method, chromatographic separation was achieved on a Zorbax SB-C18 reverse-phase column (100 mm × 3.0 mm i.d., 3.5 μm; Agilent Technologies, Santa Clara, CA, USA) maintained at 48 °C. The mobile phase consisted of a binary gradient of methanol and 0.1% (*v*/*v*) acetic acid, at a flow rate of 1 mL/min with a 5 μL injection volume. The elution profile began at 5% methanol, increased linearly to 42% over 35 min, was held isocratically at 42% for 3 min, and ended with a 7 min re-equilibration at 5% methanol. Bioactive compounds were detected using both UV and MS modes. Mass spectrometry was conducted via electrospray ionization (ESI) in negative mode, using a +3000 V capillary voltage, 60 psi nebulizer pressure, and a 12 L/min nitrogen drying gas flow at 360 °C [62]. The structural identification of the compounds was achieved using mass spectrometry in MS/MS mode. Upon positive confirmation, quantification was carried out via the UV signal. The detection UV wavelength was set to 330 nm for compounds eluting during the initial 17 min, and then switched to 370 nm until 38 min [60,61,62].

A second validated LC-MS/MS method was employed to identify additional phenolic acids and catechins. This analysis employed the same equipment and analytical column described above, with a mobile phase of methanol and 0.1% (*v*/*v*) acetic acid and an injection volume of 5 μL. The gradient elution was initiated at 3% methanol, increasing to 8% at 3 min and 20% at 8.5 min, followed by an isocratic elution at 20% until 10 min before column re-equilibration. Quantification was performed using the MS signal after positive compound identification was confirmed against the MS/MS spectra of individual reference standards [60,61,62].

The concentration of polyphenols was determined according to a calibration curve of standards ranging from 0.1 to 50 μg/mL with good linearity (R^2^ = 0.999), and the results were represented as μg of polyphenols/mL of *Coffea arabica* extract.

DataAnalysis (v5.3) and ChemStation (vB01.03) software (Agilent Inc., Santa Clara, CA, USA) were used for chromatographic data collection and processing.

### 4.3. Antioxidant Capacity (AOC)

The antioxidant activity of CA-EtAc and CA-BU fractions was evaluated using the 2,2-diphenyl-1-picrylhydrazyl (DPPH) free radical scavenging assay according to the previously reported protocol [63]. Six concentrations (1 mg/mL, 0.8 mg/mL, 0.5 mg/mL, 0.3 mg/mL, and 0.1 mg/mL) were prepared for each sub-extract to evaluate the EC_50_ parameter. First, a 0.1 mM DPPH ethanolic solution was prepared and stored at 4 °C until further use. As a standard, the ethanolic solution of ascorbic acid (1 mM in 95% EtOH) was used. A volume of 300 μL of each sample was mixed with 2.7 mL of 0.1 mM DPPH ethanol solution, and the absorbance was monitored at 517 nm for 20 min using the UviLine 9400 spectrophotometer from SI Analytics (Mainz, Germany). We expressed the results obtained as the EC_50_ value, meaning the half-maximal inhibitory concentration of the antioxidants contained in the extracts of the *Coffea arabica* green seeds, needed to scavenge the DPPH free radicals present in the test solutions by 50%. The antioxidant capacity (AOC%) was calculated by using the following equation:

$$AOC\left[\%\right]=\left[\frac{A_{DPPH}-A_{test\;sample}}{A_{DPPH}}\right]\times 100$$

where $A_{DPPH}$—the absorbance of the free radical DPPH, without the test sample, measured at 517 nm; $A_{test\;sample}$—the absorbance of each extract of *Coffea arabica* green seeds, measured at 517 nm, in the presence of DPPH free radical.

### 4.4. Antimicrobial Activity

The antimicrobial activity of the extracts was evaluated using the Disk Diffusion Method (DDM). Sterile paper disks (6 mm diameter) were impregnated with 5 μL of each extract and allowed to dry completely before being placed on the surface of agar inoculated with standardized suspensions of microorganisms. Bacteria were cultured on Mueller–Hinton agar or Mueller–Hinton agar supplemented with blood and β-NAD, and *Candida parapsilosis* on Sabouraud agar with chloramphenicol. Positive controls consisted of disks impregnated with gentamicin (10 μg/disk; 120 μg/disk for *Streptococcus pyogenes*) and fluconazole (25 μg/disk for *C. parapsilosis*), and negative controls were disks impregnated with pure solvent (dimethyl sulfoxide (DMSO) or EtOH: H_2_O 1:1). The plates were incubated at 35 °C for 24 h, and the diameters of the inhibition zones were measured in millimeters for qualitative assessment of antimicrobial activity [64,65]. All experiments were performed in triplicate, and the results are presented as the mean ± standard deviation. Compliance with EUCAST and CLSI guidelines ensures the reproducibility and comparability of results between replicates and with other similar studies [64,65].

### 4.5. Evaluation of Cell Viability

Cell viability following treatment with *Coffea arabica* sub-extracts was assessed using the Alamar Blue assay. HaCaT and MCF-7 cells were seeded in 96-well plates at an initial density of 1 × 10^4^ cells per well and incubated at 37 °C with 5% CO_2_ until reaching 80–85% confluence. The culture medium was then replaced with fresh, cell-specific medium containing increasing concentrations of the tested CA extracts (50, 100, 250, 500, and 1000 μg/mL).

After 48 h of incubation, 20 μL of Alamar Blue reagent was added to each well, followed by an additional 3 h incubation under the same conditions. Absorbance was measured at 570 nm and 600 nm using a BioTek Synergy HTX Multimode Reader (Agilent Technologies, Santa Clara, CA, USA). All experiments were performed in triplicate.

### 4.6. High-Resolution Respirometry

Mitochondrial respiratory activity was evaluated at 37 °C using a high-resolution Oxygraph-2k respirometer (Oroboros Instruments GmbH, Innsbruck, Austria). The experimental design followed a substrate–uncoupler–inhibitor titration (SUIT) protocol as described by [66]. MCF-7 cells were grown in T75 culture flasks, harvested by trypsinization, counted, and resuspended at 1 × 10^6^ cells/mL in mitochondrial respiration buffer (MIRO5: 20 mM HEPES, 20 mM taurine, 0.5 mM EGTA, 3 mM MgCl_2_, 10 mM KH_2_PO_4_, 60 mM K-lactobionate, 1 g/L BSA, and 110 mM D-sucrose, pH 7.1).

The CA-BU extract was added to the chamber immediately after the cells, at its previously determined IC_50_ value (312.24 ± 0.51 μg/mL). Digitonin (35 μg per 10^6^ cells) was used to permeabilize the cell membranes, as described by Pesta and Gnaiger [67]. The cells were first suspended in MIRO5 for 15 min prior to measurement.

Routine (mitochondrial respiration dependent on endogenous ADP) was the first respiratory rate recorded after reaching a steady state. Substrates were subsequently added following the suit protocol, consisting of: (i) digitonin and the CI (complex I) substrates (malate-M, 5 mM and glutamate-G, 10 mM)-LEAK respiratory rate (STATE2_CI_), (ii) ADP (5 mM)-complex I dependent active respiration (OXPHOS_CI_), (iii) succinate-S (10 mM), a complex II (CII) substrate, was used to determine the maximal OXPHOS capacity dependent on both complex I and complex II (OXPHOS_CI+CII_), (iv) cytochrome c (10 μM) was added to evaluate the mitochondrial membrane integrity (Cyt c), (v) oligomycin (1 μg/mL) was applied to measure LEAK respiration associated with complex I and II (State4_CI+II_), (vi) successive titrations of p-(trifluoromethoxy) phenylhydrazone carbonyl cyanide (FCCP, 1 μM/step) were used to measure the maximal respiratory capacity of the electron transport system (ETS_CI+II_), (vii) rotenone (0.5 μM) was added to determine the maximal respiratory capacity of the electron transport system complex I (ETS_CI_) dependent, (viii) finally, antimycin A (2.5 μM) was used to measure the residual oxygen consumption (ROX). All obtained values were corrected for ROX.

All data were collected using DatLab software (7.4) (Oroboros Instruments GmbH, Innsbruck, Austria) and analyzed with GraphPad Prism 5 software (GraphPad Software, Inc., San Diego, CA, USA).

### 4.7. In Vivo Evaluation of Irritation and Angiogenesis

An in vivo assessment was conducted to investigate both the potential irritative effects on mucosal-like tissues and the influence on angiogenesis of the chorioallantoic membrane (CAM) of the chick embryo. For this purpose, the in ovo CAM model was employed, a well-established platform in experimental biology and anti-angiogenic research [68,69].

Fertilized chicken eggs (*Gallus gallus domesticus*) were incubated in a humidified atmosphere at 37 °C. On day 4 of incubation, a small window was cut in the upper eggshell to allow access to the developing CAM, following an adapted protocol previously validated in our laboratory [70,71].

All experimental procedures were performed under stereomicroscopic guidance (ZEISS, Göttingen, Germany), while software for image processing, ImageJ (ImageJ Version 1.50e, https://imagej.nih.gov/ij/index.html, accessed on 5 July 2025), and GIMP (GIMP v 2.8, https://www.gimp.org/, accessed on 5 July 2025) were employed.

The protocol was performed according to ICCVAM recommendations ICCVAM Test (Method Validation Report: Current Validation Status of In Vitro Test Methods Proposed for Identifying Eye Injury Hazard Potential of Chemicals and Products—Vol 1|EURL ECVAM—TSAR https://tsar.jrc.ec.europa.eu/content/iccvam-test-method-validation-report-current-validation-status-vitro-test-methods-proposed-1 (accessed 15 July 2025), adapted to our laboratory conditions [70].

On day 10 of incubation, 300 µL of *Coffea arabica* seed extracts (CA-BU and CA-EtAc) at a concentration of 400 µg/mL were applied directly onto the CAM surface. The concentration of 400 µg/mL was selected as an intermediate value within the biologically active range identified in vitro. This dose allowed the assessment of irritation and angiogenesis under conditions where relevant antioxidant and antitumoral effects were observed, while remaining below concentrations associated with pronounced cytotoxicity.

Vascular reactions were monitored for 300 s under stereomicroscopy, and the irritation score was calculated using the equation:

$$IS=5\times \left[\frac{301-Sec\;H}{300}\right]+7\times \left[\frac{301-Sec\;L}{300}\right]+9\times \left[\frac{301-Sec\;C}{300}\right]$$

where Sec H (hemorrhage) = first appearance (in seconds) of bleeding reactions on the membrane, Sec L (lysis) = first appearance (in seconds) of lysis of the vessel on the membrane, and Sec C (coagulation) = first appearance (in seconds) of the formation of coagulation on the membrane.

Sodium lauryl sulphate (SLS) was included as a positive control, while distilled water (H_2_O) represented the negative control. The values were evaluated according to the scale established by Luepke as follows: 0–0.9—non-irritant, 1–4.9 weak irritant, 5–8.9 moderate irritant, and 9–21 strong irritant [23].

For angiogenesis evaluation, the same concentrations of CA-BU and CA-EtAc were applied within plastic rings positioned on the CAM on day 8 of incubation. The treated areas were monitored daily for 48 h under stereomicroscopy, and representative images were recorded to evaluate changes in vessel density and vascular architecture. All experiments were performed in triplicate. Results are expressed as mean ± standard deviation (SD).

### 4.8. Statistical Analysis

Statistical analyses were performed using one-way analysis of variance (ANOVA), followed by Dunnett’s post hoc test for multiple comparisons against the control group, using GraphPad Prism, version 6.0.0 (GraphPad Software, San Diego, CA, USA). Data are expressed as mean ± standard deviation (SD). Unless otherwise specified in the corresponding figure or table legend, experiments were performed in triplicate. For cell viability assays, data represent three independent experiments, each performed in triplicate. For high-resolution respirometry, data represent four independent experiments. For HET-CAM and angiogenesis assays, experiments were performed in triplicate. Statistical significance was considered at *p* < 0.05 and was reported consistently as follows: * *p* < 0.05, ** *p* < 0.01, and *** *p* < 0.001.

## 5. Conclusions

This study highlights that the butanolic (CA-BU) and ethyl acetate (CA-EtAc) fractions obtained from green *Coffea arabica* seeds exhibit distinct phytochemical profiles and biological activities, largely determined by the solvent polarity used during the extraction. Phytochemical analysis by LC–MS indicated a higher abundance of polyphenolic compounds in the CA-EtAc fraction, including catechins and chlorogenic acid derivatives, which correlated with the observed remarkable antioxidant activity (EC_50_ = 0.11 mg/mL).

In contrast, the CA-BU fraction showed comparatively higher inhibition zones in the preliminary antimicrobial screening, especially against *Staphylococcus aureus* and *Escherichia coli*, but the overall susceptibility remained low. Therefore, MIC determination is required before drawing firm conclusions regarding antimicrobial potency. In addition, the same fraction showed a significant antiproliferative effect on MCF-7 breast cancer cells. However, the close IC_50_ values obtained in MCF-7 and HaCaT cells indicate a narrow in vitro therapeutic window and limited tumor-cell selectivity. Therefore, the antiproliferative activity of the CA-BU fraction should be interpreted as preliminary mechanistic evidence rather than as direct evidence of selective anticancer potential. Bioenergetic investigations performed by high-resolution respirometry suggest that these effects may be associated, at least in part, with modulation of Complex I of the mitochondrial respiratory chain, potentially linked to the caffeoylquinic acid-rich profile of CA-BU. However, further studies using isolated compounds and recombined mixtures are required to confirm the specific constituents responsible for this effect. At the same time, both fractions demonstrated a favorable preliminary safety profile in the CAM model, with the CA-BU fraction inducing a slight reduction in the vascular density.

Overall, the results obtained contribute to expanding knowledge about the biological potential of *Coffea arabica* extracts and support the need for further investigations focused on isolating bioactive compounds and elucidating the molecular mechanisms involved in the antibacterial and anti-cancer properties, as well as validating the effects observed in more complex in vivo models. Further studies should focus on isolating the active constituents, improving tumor-cell selectivity, evaluating additional non-tumorigenic cell models, and validating the observed effects in more complex in vivo systems.

## Figures and Tables

**Table 1:** The peak values and functional groups recorded in the FT-IR spectra for butanolic and ethyl acetate *Coffea arabica* extracts.

Wave-Number (cm^−1^)	Functional Groups (CA-BU)	Bond (CA-BU)	Bond (CA-EtAc)	Functional Groups (CA-EtAc)	Wave-Number (cm^−1^)
3392.79	alcohol	OH stretch H bonded	OH stretch H bonded	alcohol	3385.07
2962.66	alkane/carboxylic acids	C-H stretch/O-H stretch	C=O stretch	carboxylic acid-conjugated	1701.22
2875.86	alkane/aldehyde	C-H stretch	C=C stretch	alkenes	1653.00
1728.22	anhydrides/aldehydes/esters	C=O stretch	C=C stretch	conjugated alkene	1600.92
1637.56	alkene/conjugated alkene	C=C stretch	C=C stretch	aromatic compounds	1550.77
1600.92	aromatic compounds/alkenes	C=C stretch	C=C stretch	aromatic compounds	1516.05
1521.84	aromatic compounds	C=C stretch	C-H bend	alkanes	1448.54
1471.69	alkanes	C-H bend	O-H bend	phenols/alcohols	1361.74
1394.53	aldehydes/carboxylic acids/phenols	C-H bend/O-H bend	C-O stretch	aromatic esters	1286.52
1375.25	alkanes/phenols	C-H bend/O-H bend	C-O stretch	ethers/alcohols	1238.30
1286.52	aromatic esters	C-O stretch	C-O stretch	alcohols (tertiary)/esters	1182.36
1122.57	secondary alcohols/aliphatic ethers	C-O stretch	C-O stretch	alcohols/carboxylic acids	1029.99
1072.42	primary alcohols	C-O stretch	C-C bend	alkenes	975.98
1039.63	alcohols/carboxylic acids	C-O stretch	C-C bend	alkenes tri-substituted	854.47
983.70	alkenes tri and di-substituted (trans)	C-C bend/=C-H bend	C-C bend	alkenes tri-substituted	813.96
920.05	alkenes	=C-H bend	C-H bend	aromatic compounds mono-substituted	744.52
866.04	alkenes	=C-H bend	C-H bend	alkynes	609.51
850.61	alkenes	=C-H bend	C-I stretch	alkyl halides	482.20
744.52	aromatic compounds mono-substituted	C-H bend	C-I stretch	alkyl halides	424.34
705.95	alkenes cis	=C-H bend	–	–	–

**Table 2:** Phenolic compounds identified in *Coffea arabica* green seed extracts by HPLC–MS analysis.

Compound	Compound Class	CA-BU (µg/mL)	CA-EtAc (µg/mL)
Chlorogenic acid	Phenolic acid	50.971 ± 2.038	48.554 ± 0.485
4-*O*-caffeoylquinic acid	Phenolic acid	7.931 ± 0.396	2.994 ± 0.209
Epicatechin	Catechin	N.D.	0.134 ± 0.012
Epigallocatechin	Catechin	N.D.	0.995 ± 0.069
Epigallocatechin gallate	Catechin	N.D.	1.68 ± 0.252

N.D.—not detected. Data are expressed as mean ± SD of three replicate measurements (*n* = 3).

**Table 3:** The initial and final AOC values (%) of CA-BU and CA-EtAC extracts at five concentrations tested as compared with the ascorbic acid ethanolic solution of 1 mM (standard), and the corresponding EC_50_ values.

Sample	Concentration (mg/mL)	Initial AOC ± SD (%)	Final AOC ± SD (%) (After 1200 s)
CA-BU	1.0	63.82 ± 6.67	81.88 ± 0.04
	0.8	53.48 ± 7.05	73.79 ± 0.04
	0.5	28.52 ± 0.12	29.63 ± 0.04
	0.3	24.58 ± 1.64	29.72 ± 0.03
	0.1	12.76 ± 0.27	14.20 ± 0.04
CA-EtAc	1.0	44.17 ± 5.85	94.41 ± 0.002
	0.8	45.94 ± 6.65	93.06 ± 0.002
	0.5	51.61 ± 7.98	91.32 ± 0.04
	0.3	48.89 ± 7.86	83.13 ± 0.04
	0.1	37.16 ± 3.28	44.37 ± 0.02
Ascorbic acid	1 mM	96.82 ± 0.006	97.36 ± 0.04

Data are expressed as mean ± SD of three independent determinations (*n* = 3). EC_50_ values were calculated by linear regression analysis based on final AOC values after 1200 s.

**Table 4:** The inhibition zone established through the disk diffusion method (DDM), after application of CA-EtAc and CA-BU fractions from green *Coffea arabica* seeds.

Fraction	Microbial Strains	DDM Inhibition Zone (mm)
CA-BU	*Streptococcus pyogenes* (Gram+)	8
	*Staphylococcus aureus* (Gram+)	12
	*Escherichia coli* (Gram−)	12
	*Pseudomonas aeruginosa* (Gram−)	7
	*Candida parapsilosis* (yeast)	11
CA-EtAc	*Streptococcus pyogenes* (Gram+)	7
	*Staphylococcus aureus* (Gram+)	8
	*Escherichia coli* (Gram−)	7
	*Pseudomonas aeruginosa* (Gram−)	7
	*Candida parapsilosis* (yeast)	11

Values represent the mean inhibition zone diameter obtained from three independent determinations (*n* = 3). The results should be interpreted as preliminary qualitative screening data; inhibition zones within the 6–15 mm range indicate low microbial susceptibility according to the applied criteria.

**Table 5:** Irritative profile of butanol (CA-BU) and ethyl acetate (CA-EtAc) fractions from *Coffea arabica* green seeds in correlation with their potential irritant effect.

Sample	Irritation Score	Type of Effect
Sodium lauryl sulfate	14.74 ± 0.36	strong irritant
H_2_O	0 ± 0	non-irritant
DMSO	0 ± 0	non-irritant
CA-BU	0 ± 0	non-irritant
CA-EtAc	0 ± 0	non-irritant

Data are expressed as mean ± SD of three independent determinations (*n* = 3). Irritation scores were interpreted according to the Luepke scale.

## Data Availability

The data supporting the findings of the study are available within the article.
